# PARTNERING WITH DEMENTIA CAREGIVERS TO DEVELOP AND REFINE THE ENACT INTERVENTION

**DOI:** 10.1093/geroni/igac059.2798

**Published:** 2022-12-20

**Authors:** Jacqueline Eaton, Sarah Neller, Moroni Fernandez Cajavilca, Julene Johnson, Lee Ellington

**Affiliations:** University of Utah, Salt Lake City, Utah, United States; University of Tennessee, Knoxville, Tennessee, United States; University of Utah, Bronx, New York, United States; University of California, San Francisco, San Francisco, California, United States; University of Utah and Huntsman Cancer Institute, Salt Lake City, Utah, United States

## Abstract

The behavioral symptoms of dementia negatively affect family caregivers. Several interventions have been developed to support caregiver needs, and evidence suggests improved outcomes when interventions actively engage caregivers. However, the strategies to implement active engagement techniques have not been sufficiently studied. Enhancing Active Caregiver Training (EnACT) is an arts-based intervention aimed at actively engaging dementia caregivers as they view and interact with participant-informed vignettes that portray caregiving experiences. We conducted a series of three iterative focus groups with dementia caregivers (n=9) to identify scenarios and refine activities for inclusion within the EnACT intervention. We analyzed the data in three cycles, using structural, descriptive, and pattern coding. From the first focus group, we coded 182 items as what went well, illustrating how caregivers resonated with video content, applied scenarios to individual experiences, and appreciated the “informative” vignettes that facilitated learning, processing, and awareness of the realities of caregiving. We coded 42 items as needing to change, including considerations for caregiver impact, confusing components, accessibility, and missing content. Participants identified eight specific vignettes that resonated and focused on the topics of respite, behavioral symptoms, validation, burnout, and communication. Intervention revisions were made based on focus group feedback, including: 1) selecting video vignettes for intervention use and 2) creating an index of vignette topics to facilitate multiple discussion pathways to align with dementia caregiver training and support groups. Partnering with dementia caregivers has the potential to make intervention activities engaging and applicable to participant needs.

